# Cactus‐Based Biopolymers: A Review on Sustainable Innovations in Edible Packaging, UV Protection, Antioxidant Films, and Industrial Applications

**DOI:** 10.1002/fsn3.71163

**Published:** 2025-12-17

**Authors:** Desye Alemu Teferi, Messenbet Geremew Kassa

**Affiliations:** ^1^ College of Agriculture, Food, Climate Science Injibara University Injibara Ethiopia

**Keywords:** biodegradable packaging, cactus mucilage, environmental sustainability, industrial applications

## Abstract

Cactus mucilage, particularly from *Opuntia ficus‐indica* , has attracted increasing scientific and industrial interest due to its unique physicochemical, structural, and functional properties. It is a heteropolysaccharide matrix enriched with uronic acids and bioactive compounds, providing high water‐binding capacity, gelling ability, pseudoplastic flow behavior, and notable film‐forming potential. These characteristics make mucilage versatile for applications in food, nutrition, and sustainable materials, although its performance remains sensitive to pH, temperature, and ionic strength. In food science and human health, cactus mucilage demonstrates safety and bioactivity. Clinical and preclinical evidence support the tolerability of daily intakes of up to 5 g of dehydrated nopal powder, while traditional diets indicate even higher safe levels. Functionally, mucilage contributes to food preservation by enhancing barriers against moisture, oxygen, and microbial contamination. Mucilage‐based films prolong shelf life through UV‐blocking and antimicrobial effects, and their functionality can be further enhanced by incorporating antioxidants, probiotics, or other natural additives. Beyond food, cactus mucilage shows promise as a biodegradable substitute for petroleum‐based plastics, aligning with sustainability initiatives. Its film‐forming, UV‐blocking, and antioxidant properties support the development of active packaging systems, while blending with other biopolymers improves strength and flexibility. Broader industrial applications include water purification through heavy metal removal, oil spill remediation, and enhancing durability and corrosion resistance in construction materials. Despite this potential, commercialization faces challenges including compositional variability, lack of standardized extraction methods, optimization of mechanical performance, and issues of scalability. In regions such as Ethiopia, valorization of cactus mucilage offers an opportunity to reduce plastic dependence, foster local industries, and create new economic opportunities. Globally, cactus‐based biopolymers contribute to Sustainable Development Goals on responsible consumption (SDG 12), climate action (SDG 13), and health (SDG 3). Continued research on safety, standardization, and cost‐effective production will be vital to fully realize its potential.

## Graphical abstract

Cactus mucilage, particularly from *Opuntia ficus‐indica* , exhibits remarkable film‐forming, UV‐blocking, antioxidant, and antimicrobial properties, making it a sustainable alternative to petroleum‐based plastics. Its applications span food preservation, edible coatings, and smart packaging systems, as well as industrial uses in construction, corrosion inhibition, and water purification. Despite promising sustainability benefits, challenges remain in scaling extraction methods, standardizing formulations, and ensuring cost‐effective commercialization.

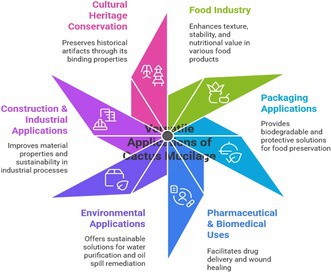

## 1 Introduction

Cactus mucilage, primarily derived from *Opuntia* species such as *O. ficus‐indica* and *O. robusta* , has recently attracted growing interest in food science, nutrition, and material sciences due to its multifunctional properties and sustainable potential. This plant‐derived hydrocolloid is rich in complex polysaccharides, dietary fiber, minerals, and bioactive compounds, which not only contribute to health‐promoting effects but also provide valuable technological and industrial applications (Dick 2019; Du Toit 2016; Slimen et al. 2016).

From a nutritional perspective, cactus mucilage contains both soluble and insoluble fibers that support digestive health, regulate intestinal function, and promote satiety (Du Toit 2016). It also provides essential minerals such as calcium, potassium, and magnesium (Dick 2019), while polyphenols and vitamins enhance antioxidant activity, reducing oxidative stress and lowering risks of chronic diseases (Patel 2015; Slimen et al. 2016). Beyond these benefits, mucilage demonstrates anti‐inflammatory, hypoglycemic, and hypocholesterolemic properties, suggesting potential applications in managing diabetes, obesity, and hypercholesterolemia (Daniloski et al. 2022; Du Toit 2016).

In food systems, cactus mucilage is recognized for its technological versatility. Its emulsifying, thickening, and stabilizing properties make it a natural alternative to synthetic additives in bakery products, sauces, dressings, and dairy alternatives (Van Rooyen et al. 2022). It can also serve as a fat replacer in formulations such as mayonnaise while maintaining desirable texture and stability (Du Toit et al. 2019). Furthermore, in gluten‐free products, mucilage not only enhances texture and structural integrity but also improves nutritional value by increasing fiber and mineral content (Dick 2019). Its high viscosity contributes to nutrient retention, supports the absorption of fat‐soluble vitamins, and improves the stability of food emulsions (Du Toit 2016).

Beyond nutrition and food formulation, cactus mucilage demonstrates unique functional properties that expand its applications in packaging and industrial sectors. Its high water retention capacity, film‐forming ability, biodegradability, and bioactivity make it an appealing alternative to synthetic polymers (Khaleel et al. 2025; Sharma et al. 2025). Mucilage‐based films possess UV‐blocking, antimicrobial, and antioxidant properties, which enhance their suitability for food and pharmaceutical packaging (Neeraj and Neetu 2024). Blending mucilage with polymers such as polyvinyl alcohol (PVA) or gelatin further improves its mechanical and barrier performance (Khaleel et al. 2025). These biodegradable films provide effective protection against oxygen, moisture, and microbial contamination while supporting active packaging strategies by incorporating antioxidants or probiotics (De Medeiros et al. 2024; Gamage et al. 2024).

The sustainability aspect further strengthens the appeal of cactus mucilage. Cacti thrive in arid and semi‐arid climates with minimal water requirements, making them highly resilient crops that support eco‐friendly biopolymer production (Gamage et al. 2024; Monteiro et al. 2023). Compared to petroleum‐based plastics, cactus‐derived biopolymers decompose naturally without leaving harmful residues, reduce landfill accumulation, and contribute to lowering greenhouse gas emissions during production (Irshad et al. 2024; Rizvi 2024; Sinha 2024). These features align with global sustainability goals and circular economy principles.

Interestingly, research has also extended to the construction sector, where cactus mucilage is incorporated into cement‐based materials to improve strength, durability, and workability. It has been shown to enhance fluidity, accelerate carbonation in lime mortars, and inhibit steel reinforcement corrosion, with efficiencies reaching up to 90% depending on concentration (Silvestre‐De‐León et al. 2021; Torres‐Acosta and González‐Calderón 2021; Velumani et al. 2023; Zhu and Huang 2024). Such applications highlight its potential in sustainable building practices.

Despite these promising benefits, challenges remain. High calcium oxalate content raises safety concerns when mucilage is consumed in excess (Du Toit 2016), while variability in composition across species, lack of standardized extraction protocols, and limited consumer awareness hinder broader adoption. Moreover, cost‐effective industrial‐scale production and property optimization are necessary for successful integration into global markets (De Medeiros et al. 2024; Teshager et al. 2024).

Overall, cactus mucilage represents a multifunctional biopolymer with applications ranging from nutrition and food technology to packaging and construction. Its abundance in regions such as Ethiopia highlights opportunities to stimulate local industries, reduce reliance on synthetic materials, and promote rural economic development. Globally, its use contributes to responsible production, climate action, and sustainable development, making it a subject of increasing scientific and industrial importance.

## 2 Methodology for Literature Selection

This review was developed through a systematic search and selection of scientific publications on cactus mucilage and its applications in biodegradable packaging and related fields. To capture both foundational studies and recent advancements, literature was retrieved from major academic databases such as Scopus, Web of Science, Science Direct, and Google Scholar, covering the period from 2020 to 2025. The search strategy employed primary keywords including “cactus mucilage,” “ *Opuntia ficus‐indica* ,” “biodegradable packaging,” “edible films,” “antioxidant activity,” “UV protection,” “industrial applications,” and “sustainable polymers,” with Boolean operators (AND, OR) used to refine and expand the scope. Studies were included if they were peer‐reviewed journal articles, book chapters, or relevant conference proceedings presenting experimental data, reviews, or applied case studies on cactus mucilage or related natural biopolymers. Only English‐language publications were considered. Exclusion criteria eliminated studies without full‐text availability, papers not directly addressing cactus mucilage or its functional properties in packaging, food, construction, or industrial applications, and non‐scientific reports or opinion pieces. In total, approximately 150 references were screened, from which 93 publications were selected for detailed review based on their methodological soundness, relevance, and contribution to understanding the functional, physicochemical, and industrial aspects of cactus mucilage. To maintain up‐to‐date coverage, most references were drawn from the last 5 years, supplemented by a limited number of earlier studies that remain highly significant.

## 3 Chemical Composition, Structure, and Rheology of Cactus Mucilage

Cactus mucilage, particularly from *Opuntia ficus‐indica* , is a highly branched heteropolysaccharide primarily composed of arabinose, galactose, rhamnose, xylose, glucose, galacturonic acid, and fucose, with its composition varying depending on species, maturity stage, and extraction method (De Medeiros et al. 2024; Elshewy et al. 2023; Makhloufi et al. 2022). Uronic acids, which can reach up to 34.5% of the total polysaccharide fraction, play a critical role in its strong gelling and thickening ability (Table 2), while arabinogalactans contribute to its high water retention and film‐forming capacity (Vargas‐Solano et al. 2022). Alongside sugars, essential minerals such as potassium, magnesium, calcium, and phosphorus enhance its structural stability and nutritional value, whereas sodium and potassium improve electrical conductivity, supporting its stability in food and pharmaceutical formulations (Vargas‐Solano et al. 2022). In addition to its polysaccharide and mineral composition, cactus mucilage is rich in bioactive compounds, including phenolics, flavonoids, and essential fatty acids, which confer antioxidant and antimicrobial properties. Total phenolic content has been reported at approximately 7.96 mg GAE/g FW, with flavonoid and flavonol levels of 3.61 and 1.47 mg QE/g FW, respectively, contributing to notable DPPH and ABTS scavenging activities (Elshewy et al. 2023) (Table 2). These bioactive constituents support the potential use of mucilage as a natural preservative in food systems, while its functional efficiency remains influenced by environmental factors and extraction techniques (Quintero‐García et al. 2021).

Structurally, cactus mucilage is distinguished by a complex, amorphous microstructure observable under scanning electron microscopy (SEM). SEM analyses reveal irregular, cracked morphologies with heterogeneous particle formations, which underpin its high water‐binding capacity and film‐forming ability. These features differentiate it from other hydrocolloids such as pectin and alginate (Van Rooyen et al. 2023). The high abundance of uronic acids in its matrix not only governs viscosity and gelling capacity but also promotes interactions with other biomolecules—an advantage in edible film preparation and composite material development.

From a rheological perspective, cactus mucilage is a highly branched heteropolysaccharide that exhibits non‐Newtonian, pseudoplastic flow behavior, where viscosity decreases with increasing shear rate, similar to xanthan gum and pectin (Du Toit et al. 2019). Its viscosity is strongly influenced by environmental factors. For instance, it is temperature dependent, with higher values observed at low temperatures (200–418 cP) and reductions at elevated temperatures (50–120 cP) (Du Toit et al. 2019). pH also plays a critical role: alkaline conditions increase viscosity (360–2400 cP), while acidic conditions reduce it (60–600 cP) (Du Toit et al. 2019). Likewise, ionic strength, particularly the presence of trivalent ions, significantly alters viscosity and gelation behavior (Du Toit et al. 2019). The formation of macromolecular aggregates in aqueous solutions further contributes to its viscoelasticity (Cárdenas et al. 1997).

The presence of acetylated low‐methoxyl pectin fragments within the mucilage matrix explains its distinctive gelling capacity. Calcium ion treatment can improve the tensile strength of mucilage‐based films, although often at the expense of elasticity (Rodríguez‐Hernández and Chavarría‐Hernández 2021; Van Rooyen et al. 2023). These combined structural and rheological features make cactus mucilage particularly suitable for food packaging applications and as a functional ingredient in nutraceutical formulations (Du Toit et al. 2019; Gheribi and Khwaldia 2019). However, the strong dependence of its properties on environmental conditions highlights the need for careful control in industrial applications (Du Toit et al. 2020).

Beyond food systems, the viscoelasticity of cactus mucilage makes it promising for industrial uses, including enhanced oil recovery, construction materials, and biopolymer composites, where its flow properties parallel those of synthetic polymers such as partially hydrolyzed polyacrylamide (HPAM) (Bourkaib et al. 2024). Nevertheless, variability in rheological performance arising from species differences, cultivation conditions, and extraction methods remains a limitation. To address this, researchers have explored blending cactus mucilage with natural and synthetic polymers, which improve mechanical stability and expand its functional applications across the food, pharmaceutical, and industrial sectors (Bourkaib et al. 2024; Van Rooyen et al. 2023).

## 4 Efficient Extraction Methods for Cactus Mucilage and Their Impact on Chemical Composition

**FIGURE 1:**
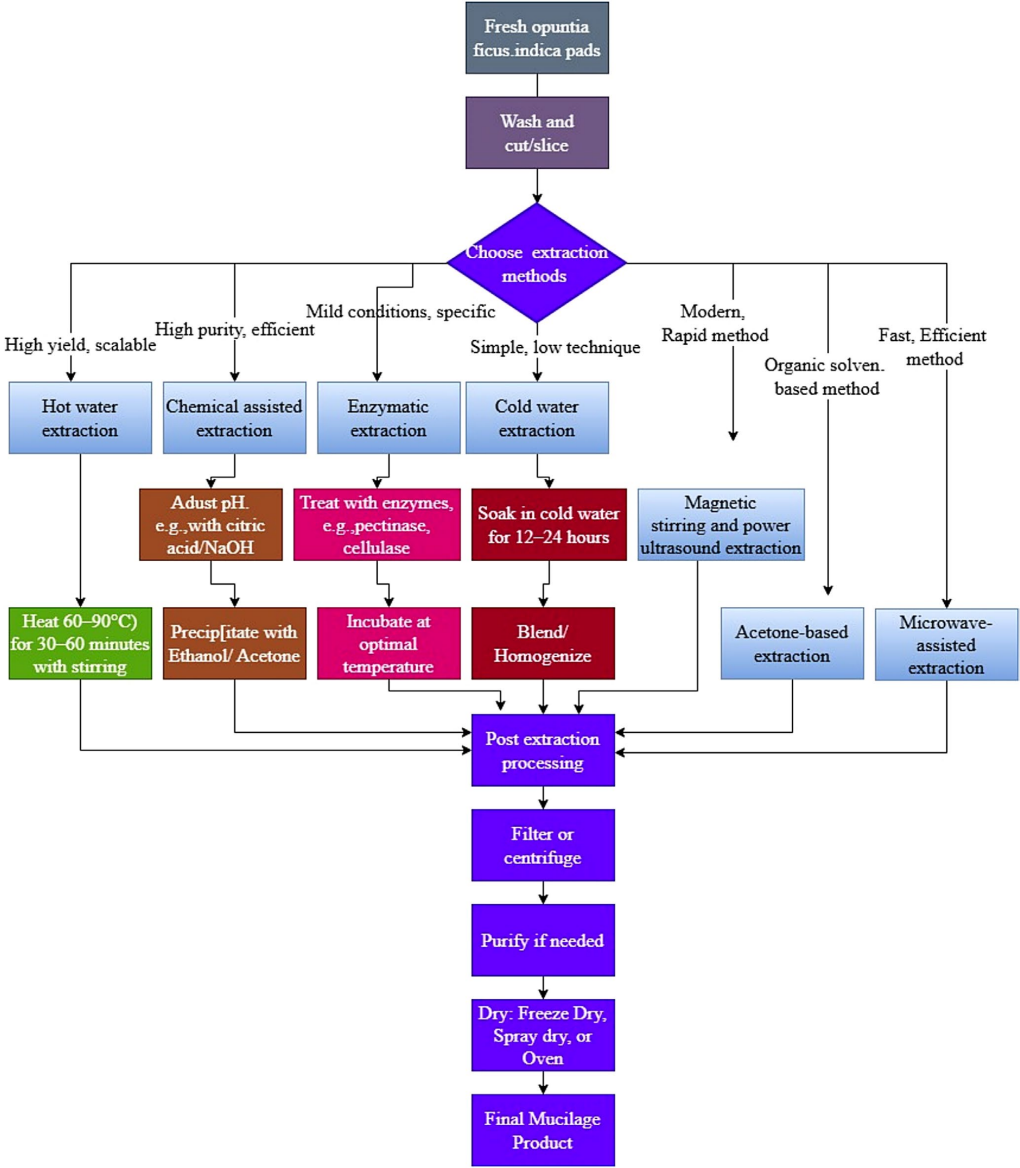
Schematic representation of the cactus mucilage extraction process.

The extraction of cactus mucilage has received increasing attention due to its wide applications in food, pharmaceutical, cosmetic, and environmental industries. The choice of extraction method significantly influences not only yield but also chemical composition and functional performance, as summarized in Table 1. Traditional techniques remain widely used for their simplicity, while recent innovations aim to improve efficiency, preserve bioactivity, and reduce environmental impact. Traditional water‐based extraction methods form the foundation for mucilage recovery. Hot water extraction, one of the earliest approaches, involves heating sliced cactus pads in water at 60°C–90°C for 30–60 min (Figure 1), followed by filtration or centrifugation. This method ensures high polysaccharide recovery but is energy‐intensive (Fernández‐Martínez et al. 2024). Cold water extraction, in contrast, involves soaking cladodes in water for 12–24 h before blending, thereby preserving sensitive bioactive compounds and maintaining mucilage integrity, though requiring longer processing times (Van Rooyen et al. 2024). Simple soaking methods have been adapted with continuous stirring or magnetic stirring to improve yield and extraction efficiency. Magnetic stirring in particular has been shown to produce mucilage with a higher content of phenolic compounds, betalains, and antioxidants, yielding up to 41.8% compared with 33.6% (Table 1) for ultrasound‐assisted extraction (Hernández‐Carranza et al. 2019). These traditional methods are well suited for small‐scale food and pharmaceutical applications but face limitations in scalability and energy efficiency.

**TABLE 1:** Summary of extraction methods of cactus mucilage and their impacts on yield.

Extraction method	Temperature (°C)	Time	Yield (%)	Notable properties	References
Hot water extraction	60–90	30–60 min	High	High polysaccharide content	Fernández‐Martínez et al. (2024)
Cold water extraction	Room temp	12–24 h	Moderate	Preserves bioactive compounds	Van Rooyen et al. (2024)
Microfiltration	N/A	Variable	Moderate	Solvent‐free, preserves proteins and polysaccharides	Fernández‐Martínez et al. (2024)
Magnetic stirring	Controlled	Variable	41.8	Rich in phenolic compounds and antioxidants	Hernández‐Carranza et al. (2019)
Power ultrasound	N/A	60 s	33.6	Faster, improves structural properties	Hernández‐Carranza et al. (2019)
Acetone‐based extraction	N/A	Variable	1.44	Suitable for pharmaceuticals	Tamboli et al. (2022)
Nonthermal hydration	Room temp	Variable	31	Enhances water‐holding and emulsifying properties	Monrroy et al. (2017)
Microwave‐assisted extraction	500 W	7 min	8.13	High protein and carbohydrate content	Felkai‐Haddache et al. (2016)
Enzyme‐assisted extraction	Variable	Variable	High	Enhances bioactive compound recovery	Kim et al. (2014)
Fresh vs. dehydrated cladodes	Variable	Variable	Similar	Dehydration increases viscosity and ash content	Quintero‐García et al. (2021)

In recent years, several advanced extraction methods have been developed to improve efficiency and maintain the functional integrity of cactus mucilage. Ultrasound‐assisted extraction enhances cell disruption, significantly reducing processing time; for example, mucilage can be released within 60 s at 30% amplitude. However, ultrasound‐derived mucilage tends to show slightly lower yields compared with magnetic stirring but exhibits superior fibril organization and film‐forming properties (Hernández‐Carranza et al. 2019). Microwave‐assisted extraction (MAE) has emerged as one of the most efficient techniques, achieving yields of up to 8.13% w/w at 500 W within 7 min (Table 1) (Felkai‐Haddache et al. 2016). MAE improves protein and carbohydrate recovery while preserving rheological functionality, making mucilage competitive with commercial gums such as gum Arabic. Enzyme‐assisted extraction further enhances recovery by using polysaccharide‐degrading enzymes to break down plant cell walls, resulting in higher sugar, phenolic, and antioxidant contents. High hydrostatic pressure (HHP) combined with enzymatic hydrolysis produces mucilage with improved solubility and stability, expanding its suitability for food and pharmaceutical formulations (Kim et al. 2014). Alcohol‐ and acetone‐based methods have also been explored, primarily for pharmaceutical applications, yielding mucilage with improved flow and surface properties despite lower overall recovery (de Andrada et al. 2024; Tamboli et al. 2022).

Extraction methods strongly influence both the chemical composition and functional performance of mucilage. For instance, dehydration of cladodes does not significantly change yield but increases protein, ash, and calcium content, thereby enhancing viscosity and altering thermal behavior (Quintero‐García et al. 2021). Similarly, microwave and ultrasound treatments increase carbohydrate and protein recovery while improving film strength, whereas enzyme‐assisted methods boost antioxidant activity by elevating polyphenol levels. Optimized combinations of temperature, enzyme activity, and treatment time have been shown to significantly improve both yield and bioactivity. Response Surface Methodology (RSM) studies indicate that a solid–liquid ratio of 1:4, extraction temperature of 90°C, and extraction time of 1 h provide optimal recovery conditions (Yang et al. 2022). These variations highlight the importance of selecting appropriate techniques depending on whether the intended application emphasizes yield, antioxidant activity, film‐forming capacity, or industrial functionality.

Environmental and economic factors also shape the suitability of different methods. Traditional hot water extractions require high energy inputs, while cold water soaking, although environmentally friendlier, demands long processing times. Advanced technologies such as MAE and ultrasound reduce processing time and solvent use but require specialized equipment and a higher initial investment. Eco‐friendly alternatives, including recycled solvents like hydro‐distilled alcohol, offer a sustainable approach to balancing efficiency with reduced environmental impact (de Andrada et al. 2024). Furthermore, agronomic and harvesting factors influence extraction outcomes. For example, non‐irrigated cactus plants produce higher mucilage content (22.2%) than irrigated plants (12.2%), while cladodes harvested in the early morning show greater mucilage yields (Costa de Sousa et al. 2022; Luna‐Zapién et al. 2023). These insights emphasize that efficient mucilage production depends not only on technological extraction methods but also on cultivation practices and resource management.

## 5 Functional Properties of Cactus Mucilage

### 5.1 Water Retention and Film‐Forming Capacity

**FIGURE 2:**
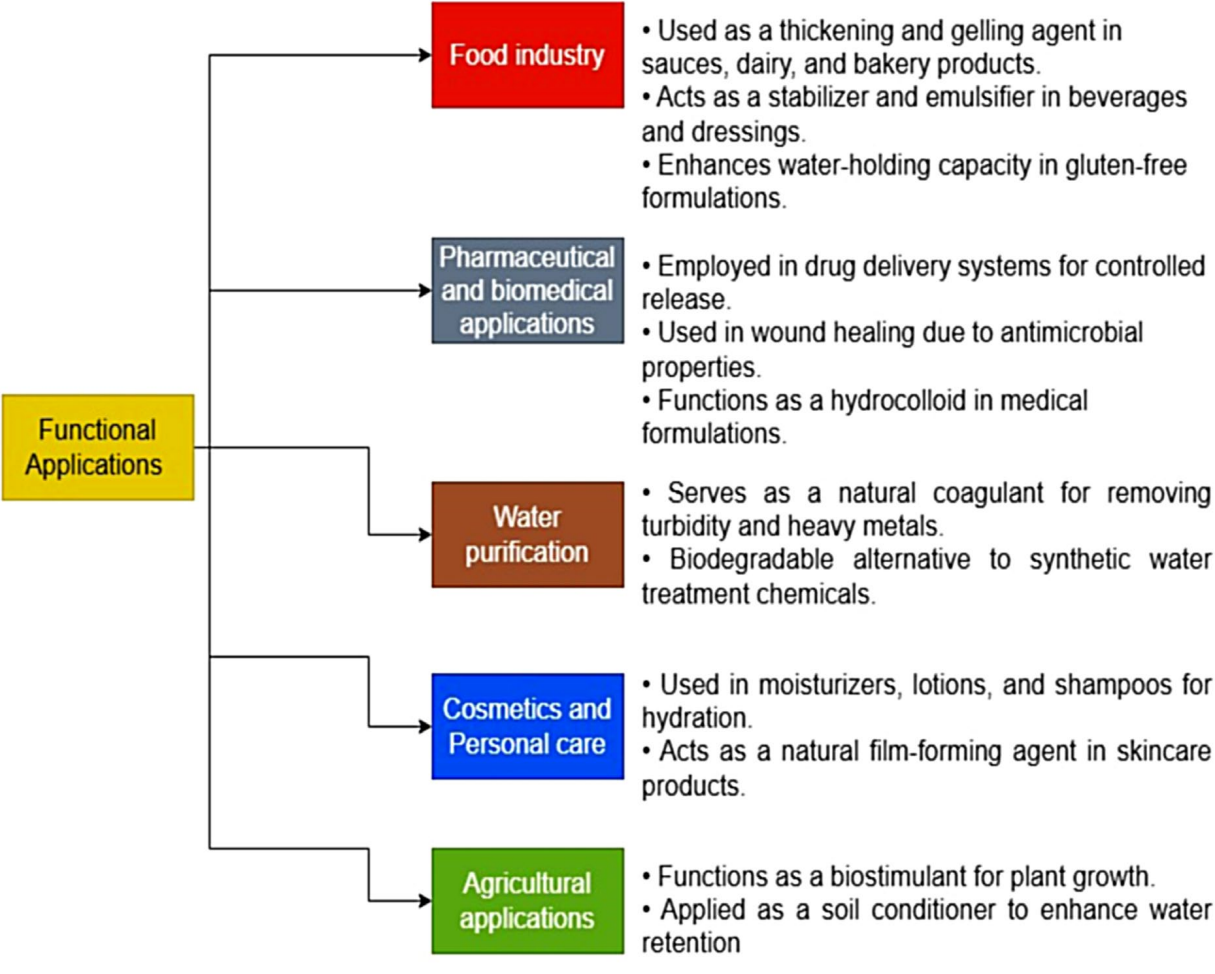
Functional applications and potential uses of cactus mucilage.

Cactus mucilage is renowned for its exceptional water retention and film‐forming capabilities, positioning it as a highly valuable biopolymer in food processing and packaging industries (Figure 2). The high concentration of uronic acids in its structure enhances its hydrophilic properties, enabling it to absorb and retain substantial amounts of water (Van Rooyen et al. 2024). When hydrated, the mucilage undergoes a viscosity increase, forming a gel‐like consistency that effectively retains moisture in food products (Du Toit 2016). Beyond its water‐binding abilities, cactus mucilage excels in forming films, making it an ideal candidate for biodegradable packaging and edible coatings. For instance, when combined with materials such as gelatin and beeswax, mucilage‐based films exhibit improved mechanical strength and serve as effective barriers against gases and moisture (Lira‐Vargas et al. 2014). Furthermore, blending mucilage with substances like styrene‐butadiene rubber latex produces flexible, hydrophobic films with low moisture content, which are particularly suitable for food packaging applications (Mannai et al. 2023). However, challenges related to the standardization of extraction methods and the enhancement of functional properties must be addressed to facilitate broader commercial adoption (De Medeiros et al. 2024).

### 5.2 Antioxidant and Antimicrobial Activities

#### 5.2.1 Antioxidant Composition and Functional Properties

Cactus mucilage is a rich source of bioactive compounds, including phenolic compounds, flavonoids, and betalains, which contribute to its antioxidant and antimicrobial properties. Studies have reported a total phenolic content of up to 1243.82 mg GAE/100 g and a total flavonoid content of 18.92 mg QE/100 g, highlighting its potent antioxidant capacity (Asiri et al. 2024). These bioactive compounds enhance the antioxidant activity of cactus‐derived films, as evidenced by assays such as ABTS and DPPH (Cenobio‐Galindo et al. 2019). Furthermore, the incorporation of cactus extracts into films improves mechanical properties, such as tensile strength and flexibility, which are critical for practical applications (Aparicio‐Fernández et al. 2018; Asiri et al. 2024).

#### 5.2.2 Stability of Antioxidants in Cactus‐Derived Films

The stability of antioxidants in cactus‐derived films is highly dependent on environmental factors such as temperature, humidity, and light. High temperatures accelerate oxidative degradation, as demonstrated by cactus seed oil stored at 40°C, which exhibited significant lipid oxidation after 6 months, whereas storage at 4°C preserved antioxidant quality over longer periods (Ettalibi et al. 2020). Relative humidity also plays a critical role; for instance, films containing L‐(+)‐ascorbic acid showed better retention at 33.3% RH compared to higher RH levels (75.2%), where degradation occurred more rapidly (De'Nobili et al. 2013).

Light exposure further impacts antioxidant stability, with high‐intensity light accelerating degradation (Mihaly Cozmuta et al. 2017). However, incorporating cactus extracts into films can enhance UV protection, thereby improving antioxidant retention (Yao et al. 2020). Additionally, cactus‐derived films with added extracts have demonstrated improved water vapor barrier properties, reducing moisture‐related degradation (Yao et al. 2020). To mitigate these challenges, encapsulation techniques and the formulation of films with protective properties have been explored to enhance the longevity and effectiveness of bioactive compounds.

#### 5.2.3 Antimicrobial and Preservative Functions

Cactus mucilage contains bioactive compounds such as polyphenols and flavonoids, which contribute to its antimicrobial and antioxidant properties (Besbes et al. 2016). These properties make it effective in inhibiting foodborne pathogens and delaying oxidative spoilage, thereby extending the shelf life of food products. For example, mucilage‐based coatings enriched with blueberry leaf extract have been used to preserve strawberries, significantly reducing microbial growth and maintaining fruit quality (Gheribi and Khwaldia 2019). The antimicrobial efficacy of cactus mucilage has also been demonstrated in seafood preservation, where it effectively inhibited spoilage microorganisms (Table 2) (Besbes et al. 2016). Additionally, the incorporation of encapsulated beetroot extract into cactus mucilage films enhances both antimicrobial and antioxidant activities. These films also exhibit pH‐sensitive color changes, enabling real‐time monitoring of food freshness (Tshamisane et al. 2025).

**TABLE 2:** Chemical composition, functional properties, and applications of cactus mucilage.

Characteristic	Observations	References
Uronic acids	34.5%	Vargas‐Solano et al. (2022)
Total sugars	0.375 mg/mL	Elshewy et al. (2023)
Flavonoids	3.61 mg QE/g FW	Elshewy et al. (2023)
Phenolic content	7.96 mg GAE/g FW	Elshewy et al. (2023)
Water retention	High moisture‐binding capacity	Van Rooyen et al. (2024)
Film formation	Forms biodegradable, flexible films	De Medeiros et al. (2024)
Antioxidant activity	DPPH scavenging: 26.15 μmol TE/g FW	Elshewy et al. (2023)
Antimicrobial properties	Effective against foodborne pathogens	Besbes et al. (2016)
Edible coatings	Extends fruit shelf life	Gheribi and Khwaldia (2019)
Biodegradable films	Reduces plastic waste	Mannai et al. (2023)
Water purification	Removes heavy metals	Gheribi and Khwaldia (2019)
Corrosion inhibition	94.5% inhibition efficiency for copper	Oulabbas et al. (2022), Zhu and Huang (2024)

Cactus‐derived biopolymers can be further enhanced with natural antimicrobial agents to improve preservative functions. For instance, the inclusion of cactus pear peel extract in sodium alginate films imparts significant antibacterial properties, which improve with higher concentrations of the extract (Asiri et al. 2024). This aligns with the broader trend of using natural antimicrobial agents, such as essential oils and plant‐derived compounds, in biopolymer‐based packaging to inhibit microbial growth and extend the shelf life of food products (Punia Bangar et al. 2021; Varghese et al. 2020). These natural additives not only provide antimicrobial activity but also contribute to the antioxidant properties of the packaging, further preserving food quality (Moeini et al. 2021, 2022).

### 5.3 UV‐Blocking Potential and Mechanical Properties

#### 5.3.1 Cactus Mucilage and Its UV‐Blocking Potential

Cactus mucilage, particularly from the *Opuntia* genus, has been extensively studied for developing edible films and coatings to preserve fruits and vegetables. These biodegradable materials can effectively prolong shelf life and maintain product quality, with potential for further enhancement through the incorporation of bioactive compounds, probiotics, and prebiotics (De Medeiros et al. 2024). Cactus pear extract, rich in betalains, can be incorporated into chitosan/polyvinyl alcohol films to enhance UV barrier properties, antioxidant activity, and antimicrobial effects (Yao et al. 2020). These natural compounds not only absorb UV radiation but also act as antioxidants and anti‐inflammatory agents, providing additional protection against UV‐induced skin damage (Saewan and Jimtaisong 2015).

##### 5.3.1.1 Mechanisms of UV Absorption

**FIGURE 3:**
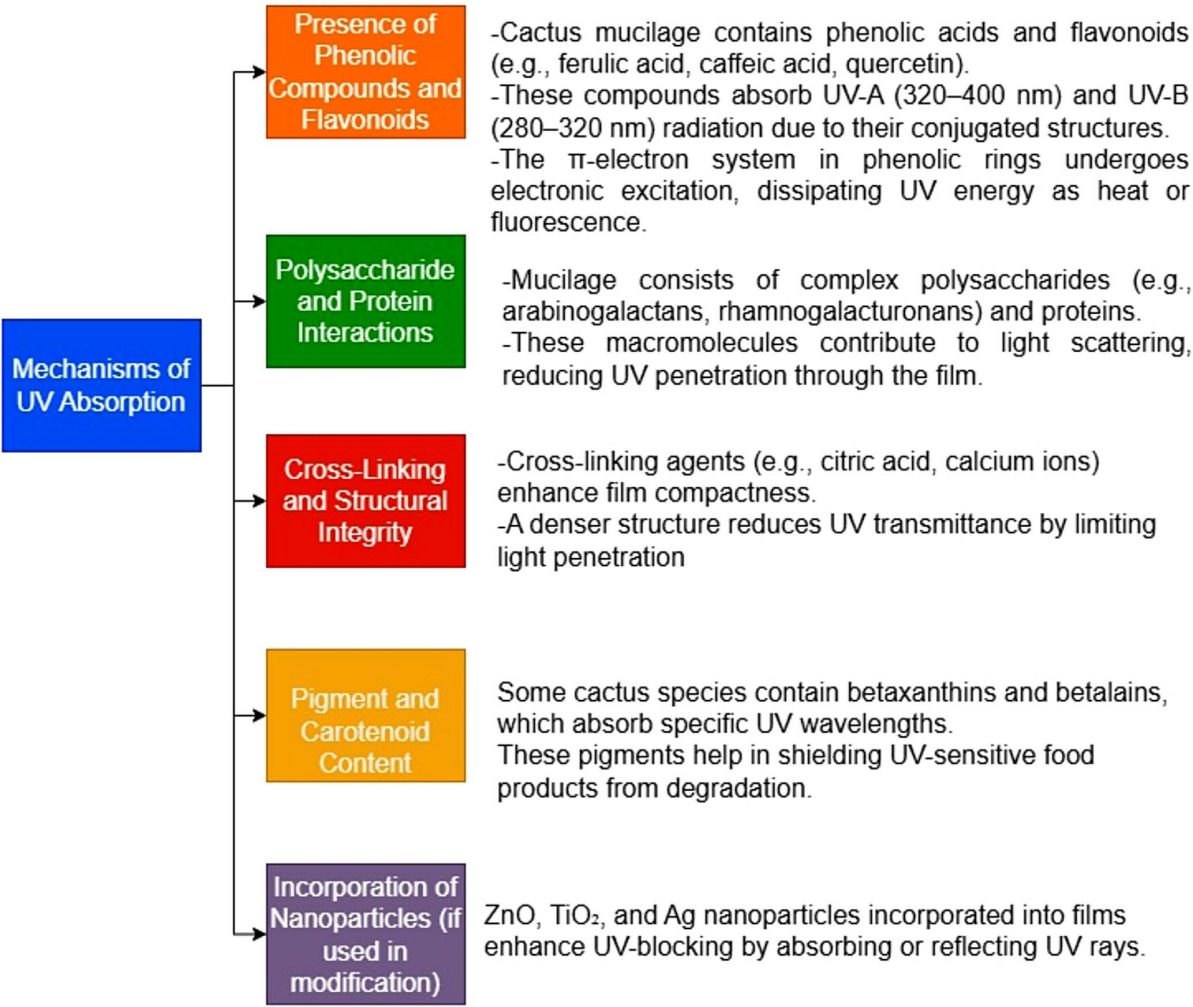
Mechanisms of UV absorption by cactus mucilage.

Cactus‐derived films exhibit strong UV‐blocking capabilities due to their bioactive composition, particularly the presence of flavonoids and phenolic compounds. The general mechanisms of UV absorption are shown in Figure 3. These compounds are known for their natural UV‐absorbing properties, which play a critical role in shielding against harmful radiation (Asiri et al. 2024; Cockell et al. 2004). Flavonoids act as effective UV filters, while phenolic compounds stabilize the film matrix and enhance UV protection. The structural morphology of the films, including thickness and transparency, also influences UV‐blocking efficiency by determining light absorption and reflection properties (Lira‐Vargas et al. 2014; Yang et al. 2019). However, environmental factors such as sunlight exposure and humidity can alter flavonoid concentrations, thereby affecting UV absorption capabilities (Cockell et al. 2004). The optimization of processing techniques is essential to maintain consistent UV‐blocking efficiency.

##### 5.3.1.2 Comparative Analysis With Synthetic UV‐Blocking Agents and Other Natural Polymers

Cactus mucilage exhibits distinct functional properties compared to other natural polymers such as pectin and alginate. Its superior water‐binding capacity enhances food texture and stability, making it a valuable ingredient in food processing (Van Rooyen et al. 2024). While pectin and alginate films generally display greater tensile strength and elasticity, the mechanical properties of cactus mucilage films vary depending on extraction methods (Van Rooyen et al. 2024). Furthermore, mucilage‐based films decompose within 40 days, offering a sustainable alternative to synthetic polymers (Mannai et al. 2023). Another unique property is their coagulation potential, as mucilage extracted from cactus peels functions as a natural coagulant, a feature not commonly found in other biopolymers (Otálora et al. 2022).

Comparing cactus mucilage with synthetic UV‐blocking agents highlights key differences in origin, efficacy, environmental impact, applications, regulatory considerations, and production costs (Table 3). Cactus mucilage is a naturally derived compound obtained from cacti, particularly rich in flavonoids that absorb UV radiation and act as antioxidants (Cockell et al. 2004; Dong et al. 2012; Saewan and Jimtaisong 2015; Yong et al. 2018). In contrast, synthetic UV‐blocking agents are chemically formulated, with organic agents absorbing UV radiation and releasing it as heat, while inorganic agents reflect and scatter UV rays. While both provide effective UV protection, synthetic agents can block nearly 100% of UVA and UVB radiation, whereas cactus mucilage has been shown to block up to 94% of UV‐B radiation (Cockell et al. 2004; Saewan and Jimtaisong 2015; Yong et al. 2018). In addition to UV protection, cactus mucilage demonstrates strong antioxidant activity by neutralizing free radicals and reducing oxidative stress. In contrast, synthetic UV‐blocking agents primarily function to block UV radiation, with only some formulations including antioxidants, often with limited efficacy (Di Napoli et al. 2024; Dong et al. 2012; Saewan and Jimtaisong 2015; Vukoje et al. 2024). This makes cactus mucilage a promising alternative for multifunctional skincare products that offer both UV protection and antioxidant benefits.

**TABLE 3:** Comparative analysis of cactus mucilage with synthetic UV‐blocking agents.

Characteristic	Cactus mucilage	Synthetic UV‐blocking agents	References
Origin and mechanism	Natural, derived from cacti; contains flavonoids that absorb UV radiation and act as antioxidants	Chemically formulated, organic agents absorb UV and release it as heat, while inorganic agents reflect and scatter UV radiation	Cockell et al. (2004), Dong et al. (2012), Saewan and Jimtaisong (2015), Yong et al. (2018)
UV‐blocking efficacy	It can block up to 94% of UV‐B radiation due to UV‐absorbing flavonoids	Some synthetic agents can block nearly 100% of UVA and UVB radiation	Cockell et al. (2004), Saewan and Jimtaisong (2015), Yong et al. (2018)
Antioxidant properties	Strong antioxidant activity; contains compounds that neutralize free radicals and reduce oxidative stress	Primarily blocks UV radiation; some formulations include antioxidants but often lack strong antioxidant properties	Vukoje et al. (2024), Saewan and Jimtaisong (2015), Dong et al. (2012), Di Napoli et al. (2024)
Sustainability and environmental impact	Eco‐friendly and sustainable; derived from low‐water‐requiring cacti	Higher environmental impact; some UV filters contribute to coral bleaching and toxicity; derived from petroleum‐based products	Cockell et al. (2004), Dong et al. (2012), Saewan and Jimtaisong (2015), Yong et al. (2018), De Medeiros et al. (2024), Rosic et al. (2023)
Biodegradability	Fully biodegradable within 40 days	Non‐biodegradable, contributes to pollution	Mannai et al. (2023)
Applications	Used in cosmetics and skincare as a natural alternative to synthetic UV filters	Used in sunscreens, textiles, and industrial coatings	Chiari‐Andréo et al. (2020), Maurya and Sen (2023)
Regulatory and safety considerations	Generally considered safe; further evaluation required for commercial applications. Non‐toxic, non‐irritating for skin	Subject to regulatory guidelines due to potential skin irritation and photosensitization. It can cause skin irritation and allergic reactions	Chiari‐Andréo et al. (2020), Saewan and Jimtaisong (2015), Yong et al. (2018), Gheribi and Khwaldia (2019)
Cost and production	Simple and cost‐effective extraction from cacti	Complex and costly chemical synthesis, though advances improve efficiency	
Future prospects	Research is needed to optimize extraction, enhance efficacy, and expand applications	Innovation focuses on reducing environmental impact and regulatory concerns, with potential for hybrid formulations	Cockell et al. (2004), Vukoje et al. (2024), Saewan and Jimtaisong (2015), Yong et al. (2018)

Sustainability and environmental impact further distinguish these two options. Cactus mucilage is highly sustainable, derived from drought‐resistant plants that require minimal water (Cockell et al. 2004; Dong et al. 2012; Saewan and Jimtaisong 2015; Yong et al. 2018; De Medeiros et al. 2024; Rosic et al. 2023). In contrast, synthetic UV‐blocking agents have a higher environmental footprint, with some contributing to coral bleaching and marine toxicity. Additionally, cactus mucilage is fully biodegradable within 40 days, whereas synthetic agents are non‐biodegradable and contribute to long‐term pollution (Mannai et al. 2023). The applications of these agents differ as well, with cactus mucilage gaining popularity in cosmetics and skincare as a natural UV filter alternative, while synthetic agents are widely used in sunscreens, textiles, and industrial coatings (Chiari‐Andréo et al. 2020; Maurya and Sen 2023). Regulatory and safety considerations also vary, as cactus mucilage is generally recognized as safe, with non‐toxic and non‐irritating properties, though further evaluation is needed for commercial use. Synthetic agents, on the other hand, must comply with strict regulatory guidelines due to concerns over skin irritation and photosensitization (Chiari‐Andréo et al. 2020, 2021; Saewan and Jimtaisong 2015; Yong et al. 2018; Gheribi and Khwaldia 2019).

Cost and production efficiency further distinguish these two approaches. Cactus mucilage is simpler and more cost‐effective to extract compared to the complex and expensive synthesis of synthetic UV filters. However, advances in chemical engineering are improving the efficiency of synthetic production methods (Dong et al. 2012). Looking ahead, research on cactus mucilage should focus on optimizing extraction techniques, enhancing UV‐blocking efficacy, and expanding its applications. Meanwhile, innovation in synthetic UV‐blocking agents is centered on reducing environmental impact and addressing regulatory concerns, with potential for hybrid formulations that combine natural and synthetic components for improved sustainability (Cockell et al. 2004; Dong et al. 2012; Vukoje et al. 2024; Saewan and Jimtaisong 2015; Yong et al. 2018).

## 6 Applications of Cactus Mucilage in Film Formation and Its Properties

Cactus mucilage, particularly from *Opuntia ficus‐indica* , has gained increasing attention as a sustainable biopolymer for food packaging due to its eco‐friendly, biodegradable nature and functional film‐forming properties. Its polysaccharide‐rich composition, consisting mainly of arabinose, galactose, and uronic acids, provides a structural foundation for edible films and coatings that can preserve food quality and extend shelf life (De Medeiros et al. 2024; Gheribi and Khwaldia 2019; Van Rooyen et al. 2024). The efficiency and performance of cactus mucilage films largely depend on the extraction methods employed, the processing steps\used for film casting, and the incorporation of additives or reinforcements that tailor mechanical and barrier characteristics.

Film production begins with the careful selection of fresh cactus pads, especially the cladodes of *Opuntia ficus‐indica* , which are known for their high mucilage content. The pads are washed, cut into smaller pieces, and subjected to extraction using various methods (Costa de Sousa et al. 2022; De Medeiros et al. 2024).

Traditional techniques include hot water extraction, where cactus segments are boiled at 60°C–90°C for 30–60 min, and cold water extraction, which involves soaking cladodes for 12–24 h, followed by blending (De Medeiros et al. 2024; Teshager et al. 2024). Advanced methods such as microfiltration and microwave‐assisted extraction have been developed to improve yield and preserve polysaccharide integrity (Fernández‐Martínez et al. 2024; Teshager et al. 2024). Once extracted, mucilage is filtered or centrifuged to remove solids and then dried using spray‐drying or freeze‐drying into a powdered form, which is more stable for film preparation (Costa de Sousa et al. 2022; Teshager et al. 2024). For film formation, the powdered mucilage is dissolved in water (1%–5%), the pH is adjusted to improve film‐forming capacity, and plasticizers such as glycerol are added to enhance flexibility. The solution is then cast onto flat surfaces or molds and dried at controlled temperatures (40°C–60°C) to form thin films of desired thickness (De Medeiros et al. 2024; Van Rooyen et al. 2024).

Significant progress has been made in enhancing the mechanical and barrier properties of cactus mucilage films. Early modifications involved the addition of glycerol and reinforcing agents such as kaolin to improve flexibility and structural integrity (Teshager et al. 2024; Van Rooyen et al. 2023). Cross‐linking with citric acid at concentrations of about 0.5% further increased tensile strength and crystallinity (Kumar et al. 2024). Blending mucilage with other biopolymers, such as agar and gelatin, improved thermal stability, UV‐light protection, and water vapor resistance (Makhloufi et al. 2022), while mucilage–PVA composites (80:20 ratio) achieved a 165% increase in tensile strength and superior UV–vis barrier properties (Gheribi and Khwaldia 2019). Incorporation of gelatin and beeswax enhanced transparency and reduced water vapor permeability, making the films more suitable for postharvest conservation (Lira‐Vargas et al. 2014). Bioactive enrichment with phenolics, flavonoids, and essential oils has provided films with antimicrobial functionality; for example, mucilage films with blueberry leaf extract or thymol oil reduced microbial spoilage in strawberries and blackberries, extending their shelf life (Moeini et al. 2022). Efforts to incorporate probiotics also highlighted the potential of these films as carriers of functional ingredients, although further optimization is required to ensure microbial survival (Todhanakasem et al. 2022). More recent strategies, such as calcium ion treatments with calcium lactate and calcium chloride, increased tensile strength up to 12.91 MPa, while nanofillers such as acid‐leached kaolin and cellulose nanofibers improved flexibility, moisture resistance, and oxygen barrier properties (de Andrada et al. 2024; Tshamisane et al. 2025). Nevertheless, challenges such as water sensitivity, linked to the hydrophilic nature of additives like gelatin, continue to limit broader applications, and standardization of formulations remains a key research priority (Berry III et al. 2007).

Applications of cactus mucilage in film formation extend beyond simple food packaging. Its mechanical strength and barrier properties, particularly low water vapor transmission rates (≤ 10.6 g h^−1^ m^−2^ when blended with agar), make it suitable for preserving fresh produce by reducing spoilage and extending shelf life (Makhloufi et al. 2022). Blends with PVA and CNFs have shown promise in creating films with high tensile strength, flexibility, and excellent oxygen and CO_2_ barrier properties, making them ideal for packaging perishable fruits and vegetables (Gheribi and Khwaldia 2019; Tshamisane et al. 2025). The incorporation of beeswax has allowed the development of oil‐resistant films suitable for packaging oily foods (Lira‐Vargas et al. 2014). Beyond food, cactus mucilage‐based films are being explored in pharmaceuticals for controlled drug release, wound healing applications, and in cosmetics due to their biocompatibility and biodegradability (Martín et al. 2024; Van Rooyen et al. 2024).

**FIGURE 4:**
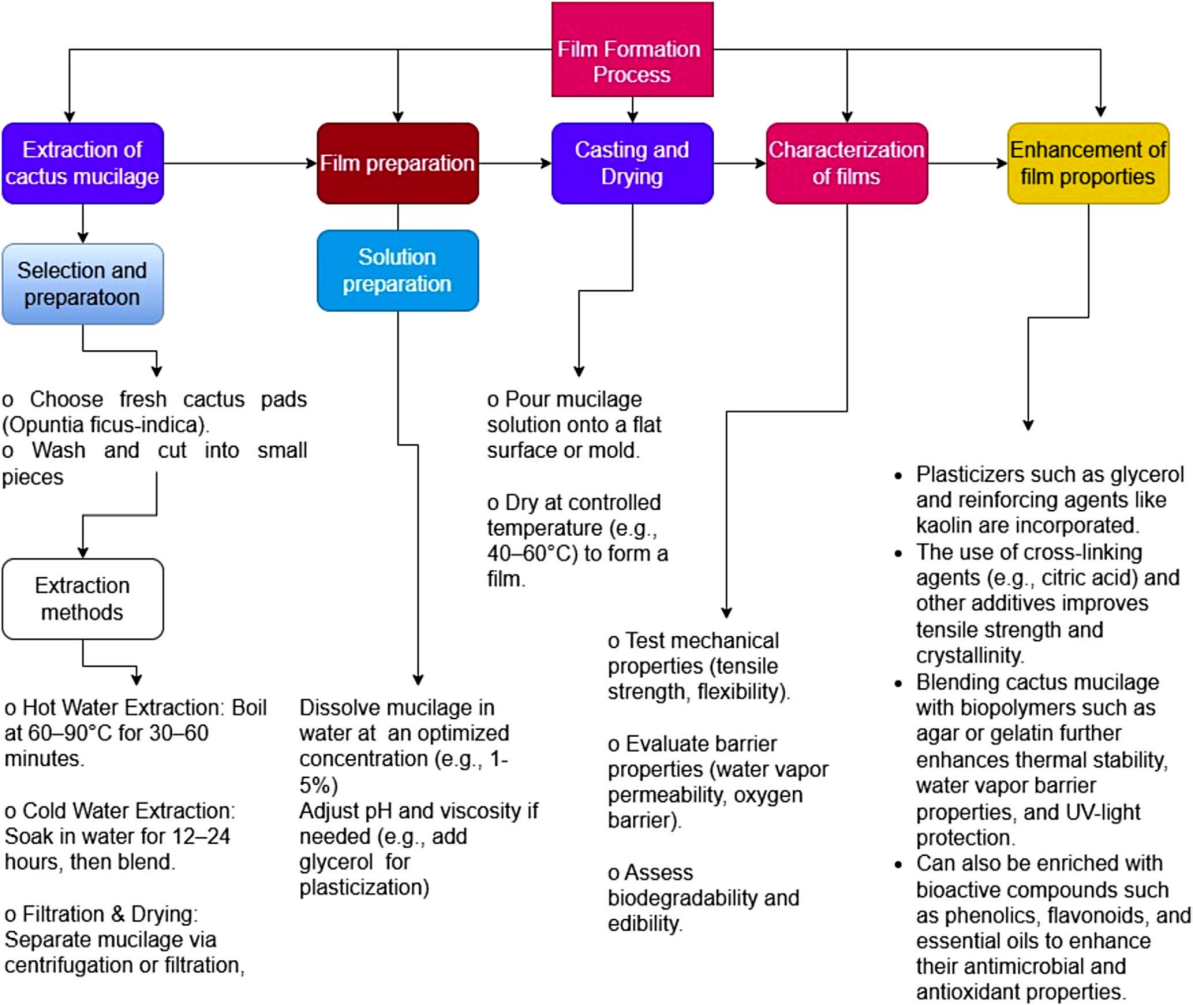
Flow diagram for film formation using cactus mucilage.

From an environmental and industrial perspective, cactus‐derived films align well with circular economy principles since they are biodegradable, renewable, and reduce reliance on petroleum‐based plastics. They minimize plastic waste and prevent harmful chemical leaching into food systems (Oudir et al. 2023). However, commercialization faces challenges, primarily in scaling production and ensuring consistency of extraction methods and film properties (Teshager et al. 2024). Standardization of processes and optimization of film formulations remain critical to achieving industrial adoption. With ongoing improvements in material blending, nanostructuring, and the incorporation of bioactive compounds, cactus mucilage‐based films hold strong potential as sustainable packaging and biomedical materials in the future. The film formation process using cactus mucilage is illustrated in Figure 4.

### 6.1 Cactus Mucilage in Sustainable Material Applications

**FIGURE 5:**
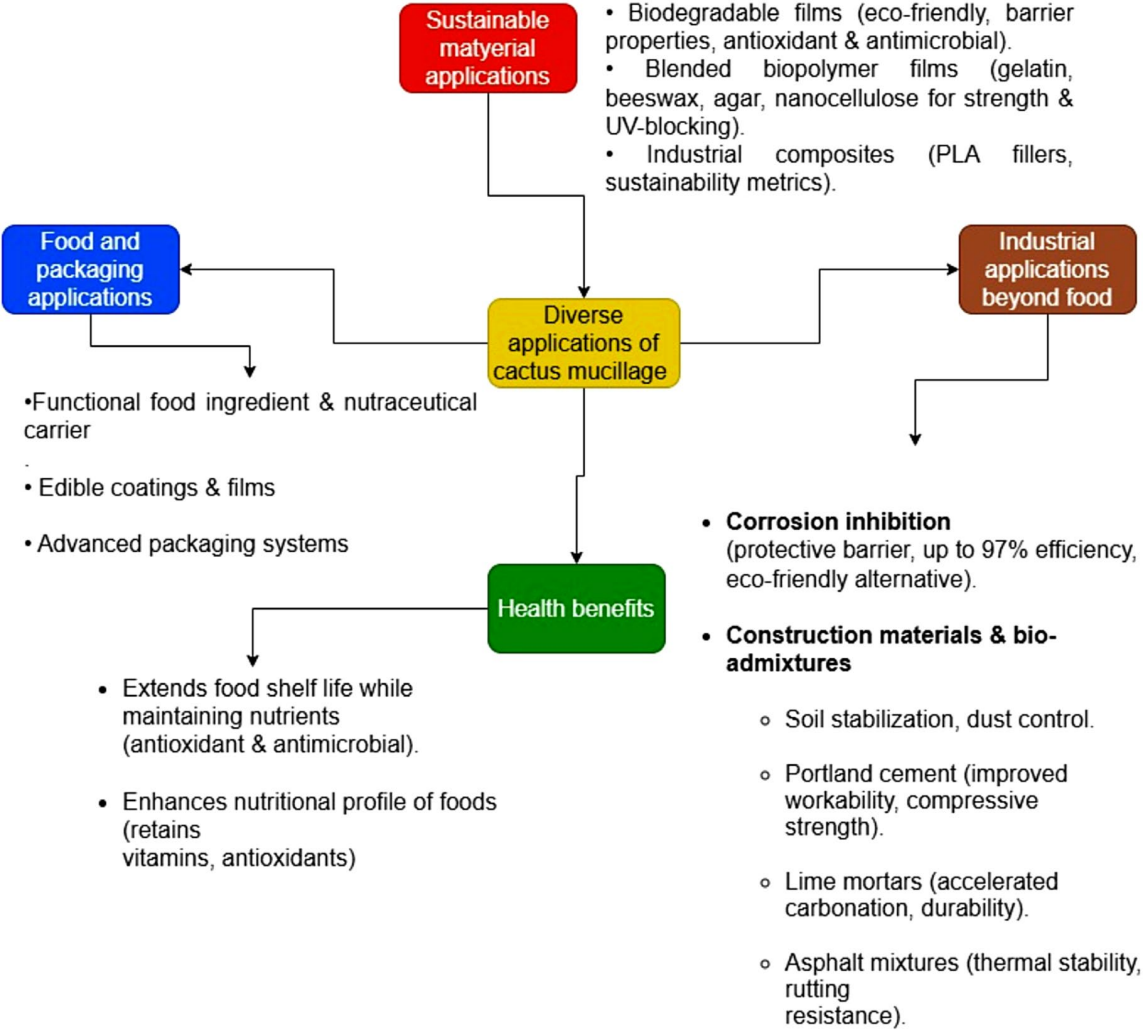
Diverse applications of cactus mucilage.

Cactus mucilage, particularly from species such as *Opuntia ficus‐indica* , has emerged as a highly promising natural polymer for developing sustainable materials (Figure 5). Its polysaccharide‐rich composition grants it exceptional film‐forming ability, biodegradability, and bioactive properties, positioning it as a viable alternative to conventional petroleum‐based plastics. From an environmental perspective, films derived from cactus mucilage are fully biodegradable, thereby mitigating the persistent issue of plastic waste and reducing risks associated with chemical migration into food and the environment. The production of these materials aligns with circular economy principles by valorizing a renewable resource and providing a sustainable source for packaging applications (De Medeiros et al. 2024; Khaleel et al. 2025; Sharma et al. 2025).

The functional efficacy of cactus mucilage films stems from their inherent characteristics. They provide a competent barrier against gases like oxygen and carbon dioxide, as well as moisture, which is crucial for food preservation. Furthermore, their intrinsic antioxidant and antimicrobial activities contribute directly to enhanced food safety and shelf‐life extension (Gheribi and Khwaldia 2019; Kumar et al. 2024) (Figure 5). To overcome limitations in mechanical strength and water resistance, research has focused on blending mucilage with other biopolymers. Integrations with gelatin, beeswax, and agar have been shown to significantly improve tensile strength, flexibility, and resistance to water vapor, making the composite films suitable for practical applications like postharvest conservation (Lira‐Vargas et al. 2014). The addition of nanomaterials such as nanocellulose further enhances barrier functions and mechanical properties, creating films with a higher Young's modulus and improved UV‐blocking capabilities (Makhloufi et al. 2022; Tshamisane et al. 2025).

Despite its significant potential, the commercial adoption of cactus mucilage requires overcoming substantial challenges. The transition from laboratory research to industrial applications hinges on establishing cost‐effective extraction methods, achieving consistent formulations to combat natural variability, and developing industrial‐scale curing processes. Advances in nanotechnology, polymer blending, and standardized production protocols are therefore critical for its future commercialization (Alizadeh‐Sani et al. 2020).

### 6.2 Diverse Industrial Applications

The application of cactus mucilage extends well beyond the food industry, demonstrating remarkable versatility. In the field of cultural heritage conservation, it serves as a traditional and effective binder for paints and coatings used in the preservation of historical artifacts (D'Agostino et al. 2023). Within the energy sector, its unique rheological properties are leveraged in Enhanced Oil Recovery (EOR) processes to improve the extraction of oil from reservoirs (Bourkaib et al. 2024). This wide range of applications underscores the material's multifunctional nature and its potential to contribute to sustainability across various sectors.

In the food sector, cactus mucilage has gained considerable attention due to its versatile functional properties, which make it a valuable ingredient for improving product texture, stability, and nutritional profile.

#### 6.2.1 Functional Food Ingredient and Nutraceuticals Carrier

A primary application is in the production of gluten‐free foods. The mucilage's rheological properties and ability to retain moisture enhance the mouthfeel, softness, and overall shelf life of products like bread, crackers, and cakes, which are often challenging to formulate without gluten (Salem et al. 2024). Beyond its textural role, cactus mucilage is being investigated as a bioactive ingredient and carrier in nutraceuticals. It effectively encapsulates sensitive compounds, including vitamins, minerals, and plant extracts, protecting them from degradation and thereby preserving their stability and efficacy for enhanced health benefits (Tabio‐García et al. 2023). Its inherent antioxidant properties further enrich the nutritional profile of functional foods.

#### 6.2.2 Edible Coatings and Films for Food Preservation

One of the most widely explored applications is the use of cactus mucilage as an edible coating for fresh produce, meat, and seafood. Applied directly to foods like strawberries, tomatoes, and bell peppers, these coatings form a protective barrier that reduces moisture loss, delays ripening, and suppresses microbial growth, effectively extending shelf life (De Medeiros et al. 2024; Kumar et al. 2024). The functionality of these coatings can be significantly enhanced by enriching them with bioactive compounds such as blueberry leaf extract or essential oils, which have demonstrated notable success in reducing spoilage in berries and fresh‐cut fruits (Gheribi and Khwaldia 2019). In meat and seafood systems, mucilage coatings act as effective oxidation inhibitors, delaying lipid peroxidation and maintaining product quality (De Medeiros et al. 2024).

#### 6.2.3 Advanced Packaging Systems

The application of cactus mucilage has advanced beyond simple edible coatings to more sophisticated packaging systems that actively interact with food products. In active packaging, the natural antioxidant and antimicrobial properties of mucilage are further enhanced by incorporating compounds such as phenolics, flavonoids, or essential oils. For example, mucilage‐based films enriched with thymol oil or cactus pear extract have shown significantly improved antimicrobial efficacy, leading to an extended shelf life of packaged foods (Punia Bangar et al. 2021). Another emerging direction is the development of probiotic‐enriched mucilage films, where the mucilage matrix serves as a carrier for beneficial microorganisms; however, further optimization is needed to maintain probiotic viability during storage and use (Todhanakasem et al. 2022). In contrast, intelligent packaging represents a more cutting‐edge innovation, in which nanomaterials such as nanocellulose and halloysite nanotubes are incorporated into mucilage films. These functional materials enable controlled release of active compounds and heightened responsiveness to environmental changes, such as shifts in pH or temperature, allowing the films to act as real‐time freshness indicators while simultaneously prolonging food quality and safety (Makhloufi et al. 2022). Overall, cactus mucilage‐based films and coatings exhibit remarkable versatility, ranging from edible and active to intelligent and biodegradable systems. When combined with other biopolymers or natural additives, they deliver enhanced performance compared with standalone mucilage, underscoring their potential as strong candidates for next‐generation sustainable packaging solutions (Table 4).

**TABLE 4:** Comprehensive applications of cactus mucilage in packaging and material science.

Application category	Primary function	Key components and additives	Major benefits	Current challenges and considerations	References
Edible coatings	Form a protective, biodegradable barrier on food surfaces to extend shelf life	Mucilage base enriched with blueberry leaf extract, essential oils (e.g., thymol)	Reduces moisture loss, delays ripening, suppresses microbial growth, and inhibits oxidation in meat/seafood	Adherence to different food surfaces, sensory impact on taste/odor, and scaling application processes	De Medeiros et al. (2024), Kumar et al. (2024), Gheribi and Khwaldia (2019)
Active packaging	Actively interact with the food to improve preservation and safety	Mucilage matrix with natural additives: phenolics, flavonoids, essential oils, probiotics	Inhibits microbial growth, prevents lipid oxidation, and extends shelf life, with potential probiotic delivery	Ensuring stability and efficacy of active compounds while maintaining probiotic viability during storage	Punia Bangar et al. (2021), Asiri et al. (2024), Todhanakasem et al. (2022)
Intelligent (Smart) packaging	Monitor food freshness and respond to environmental changes	Mucilage integrated with nanomaterials: nanocellulose, halloysite nanotubes	Provides real‐time spoilage indicators (e.g., pH change), enables controlled release of active compounds	High cost of nanomaterials, ensuring accuracy and reliability of indicators, and regulatory approval	Makhloufi et al. (2022)
Blended biopolymer films	Enhance mechanical and barrier properties for robust packaging	Mucilage blended with gelatin, beeswax, agar, or reinforced with nanocellulose	Increases tensile strength, flexibility, UV‐blocking, and water vapor resistance	Optimizing blend ratios for consistency, cost of secondary biopolymers/nanomaterials	Lira‐Vargas et al. (2014), Makhloufi et al. (2022), Tshamisane et al. (2025)
Industrial composites (non‐food)	Valorize cactus waste as a sustainable additive in material science	Cactus biomass or mucilage as a filler in matrices like PLA (Polylactic Acid)	Improves sustainability metrics of composites, utilizes waste products, and leverages existing manufacturing	Achieving consistent material properties from a variable waste stream and integration into industrial processes	Botta et al. (2025), Oudir et al. (2023)
Nonpackaging applications	Leverage functional properties in other sectors	Pure or minimally processed mucilage	Cultural conservation: Binder for paints/coatings. Energy: Enhances oil recovery (EOR)	Developing standardized grades for different industries and establishing supply chains	D'Agostino et al. (2023), Bourkaib et al. (2024)

### 6.3 Current Status of Commercial Implementation

Despite a significant expansion in academic research over the past decade, focusing on extraction methods, formulation, and testing, the commercial implementation of cactus mucilage‐based packaging remains in its nascent stages. The technology is predominantly confined to laboratory and pilot‐scale projects, with only a limited number of prototype efforts advancing toward commercialization (Alves et al. 2025; Van Rooyen et al. 2024).

Current industry‐oriented development has followed two pragmatic pathways. The first involves creating value‐added composites, where cactus residues or mucilage are incorporated as functional fillers or additives into established biopolymer matrices, such as Polylactic Acid (PLA) (Figure 5). This approach leverages existing processing infrastructure while improving the sustainability profile of the final product. The second pathway focuses on optimizing standalone mucilage films and coatings at a lab scale before attempting small pilot trials for specific barrier or active functions (Botta et al. 2025; Oudir et al. 2023).

Several formidable barriers impede widespread industrial adoption. These include the inherent variability in mucilage composition due to species, harvest time, and growing conditions; a lack of standardized, cost‐effective, and scalable extraction and drying processes; and performance limitations in mechanical strength and water resistance compared to synthetic polymers. Furthermore, economic competitiveness, regulatory clearance for food contact, consumer acceptance, and the development of a reliable supply chain for consistent raw material sourcing are critical hurdles that remain largely unresolved. For meaningful commercialization to occur, future work must prioritize solving these standardization and scale‐up problems, complemented by comprehensive life‐cycle and cost–benefit analyses (Oudir et al. 2023; Teshager et al. 2024).

## 7 Industrial and Environmental Applications Beyond Food Packaging

### 7.1 Corrosion Inhibition in Industrial Settings

Cactus mucilage has emerged as a high‐performance, eco‐friendly corrosion inhibitor, particularly in acidic environments. Its effectiveness stems from its ability to form a hydrophobic barrier on metal surfaces, blocking corrosive agents (Figure 5). Studies report up to 94.5% inhibition efficiency for copper corrosion in sulfuric acid environments at optimal concentrations, with adsorption mechanisms following the Langmuir isotherm model (Table 2) (Zhu and Huang 2024). The mucilage acts as a mixed‐type inhibitor, with strong adsorption characteristics on metal surfaces, following the Langmuir isotherm model (Oulabbas et al. 2022; Zhu and Huang 2024).

The inhibition process involves three stages: (1) protective film formation, (2) adsorption of mucilage molecules to neutralize surface charges, and (3) stabilization of the metal interface (Oulabbas et al. 2022). The inhibition efficiency of cactus mucilage has been reported to exceed 90% for various metals, including copper and mild steel, with optimal concentrations yielding efficiencies as high as 97.7% (Zhu and Huang 2024; Oulabbas et al. 2022; Özkır 2023).

Moreover, cactus mucilage offers a sustainable alternative to traditional chemical inhibitors, which often have detrimental environmental impacts. For instance, *Mammillaria prolifera* extract achieved > 90% anti‐corrosive performance in 1.0 M HCl, validated by electrochemical tests and FE‐SEM imaging (Özkır 2023).

### 7.2 Advancements in Construction Materials and Bio‐Admixtures

Cactus mucilage has emerged as a transformative bio‐admixture in the construction industry, enhancing the mechanical, rheological, and durability properties of cement‐based materials (Figure 5). Early studies highlighted its potential as a natural coagulant for stabilizing soil and controlling dust on construction sites, offering an eco‐friendly alternative to synthetic binders (Alcantar et al. 2012). Innovations expanded to 3D‐printed concrete applications, where its rheological properties facilitated precise layering and reduced material waste (Berry III et al. 2007). Subsequent research demonstrated that incorporating mucilage into Portland cement mixtures improves workability and compressive strength. For instance, concrete cured at 60°C exhibited a 20% increase in compressive strength due to refined pore structure and reduced cracking (Watters and Bernhardt 2018).

Further advancements revealed that cactus mucilage enhances adhesion in mortar applications, making it ideal for plaster and repair work (León‐Martínez et al. 2021). Its polysaccharide‐rich composition also promotes accelerated carbonation in lime mortars, catalyzing the formation of stable calcium carbonate and boosting durability (León‐Martínez et al. 2021). In steel‐reinforced mortar exposed to CO_2_‐rich environments, mucilage demonstrated corrosion inhibition efficiencies of 40%–90%, depending on concentration, thereby extending structural lifespan (Torres‐Acosta and González‐Calderón 2021). Recent studies reported a 30% increase in concrete durability at optimal mucilage concentrations, attributed to improved fluidity and compaction (Velumani et al. 2023).

The application of cactus mucilage has extended to asphalt mixtures, where it enhances thermal stability and resistance to rutting, prolonging pavement lifespan (Oudir et al. 2023). Despite these benefits, challenges such as variability in mucilage composition driven by species‐specific differences and extraction methods pose hurdles for consistent performance (De Medeiros et al. 2024; Van Rooyen et al. 2023). Recent efforts emphasize the need for standardized protocols and lifecycle assessments to evaluate long‐term environmental and economic impacts (Chavez et al. 2022; Teshager et al. 2024). With optimized formulations, cactus mucilage could reduce reliance on synthetic additives, aligning with green building practices and lowering the carbon footprint of construction materials (León‐Martínez and Cano‐Barrita 2024; Zhu and Huang 2024).

### 7.3 Development of Biodegradable Single‐Use Products

The use of cactus mucilage in biodegradable single‐use products has evolved significantly over recent years, driven by the need for sustainable alternatives to petroleum‐based plastics. Early research demonstrated the potential of mucilage's natural polysaccharide structure to form durable yet compostable films, with studies showing that blending mucilage with glycerol and kaolin yielded materials with 6.74 MPa tensile strength and flexibility (Gheribi and Khwaldia 2019; Teshager et al. 2024). Subsequent advancements revealed that these films decompose within 40 days under natural conditions, minimizing environmental pollution (Mannai et al. 2023). By 2022, cactus mucilage‐based shopping bags were shown to rival low‐density polyethylene (LDPE) in tensile strength while degrading rapidly in soil, marking a critical milestone in practical applications (Chavez et al. 2022).

Further innovations focused on aligning mucilage‐derived materials with circular economy principles, emphasizing their non‐toxic, renewable nature and reduced chemical leaching (Oudir et al. 2023). However, challenges such as scalability, cost‐effectiveness, and variability in extraction methods hindered large‐scale commercialization (De Medeiros et al. 2024; Van Rooyen et al. 2023). Recent breakthroughs addressed these limitations by blending mucilage with polyvinyl alcohol (PVA) and cellulose nanofibers, enhancing water resistance and durability (Tshamisane et al. 2025). Collaborative efforts between researchers and industry stakeholders are now pivotal to standardizing production processes and overcoming regulatory barriers, ensuring cactus mucilage becomes a mainstream biodegradable material (Chavez et al. 2022; Teshager et al. 2024). With continued optimization, these innovations promise to revolutionize single‐use products, offering eco‐friendly solutions that balance performance and sustainability.

### 7.4 Environmental Remediation and Water Purification

Beyond industrial uses, cactus mucilage plays a role in environmental sustainability. As a natural coagulant, it effectively removes heavy metals and contaminants from water (Table 2), offering a green alternative to synthetic agents (Gheribi and Khwaldia 2019). Its oil‐absorbing properties also make it valuable for eco‐friendly oil spill cleanup (Alcantar et al. 2012). These applications highlight its dual role in pollution mitigation and resource conservation, underscoring its potential in circular economy frameworks.

Additionally, cactus mucilage has been explored as an eco‐friendly corrosion inhibitor for metals such as copper, with water‐based extraction methods yielding mucilage with high inhibition efficiency (Zhu and Huang 2024). In the food industry, cactus mucilage serves as a natural hydrocolloid, improving moisture retention and sensory characteristics in gluten‐free products such as flatbread (Salem et al. 2024). Its potential as a biodegradable alternative to petroleum‐based packaging materials is being actively explored, addressing growing environmental concerns (Teshager et al. 2024). The mucilage's ability to enhance the functional properties of food products while offering a sustainable packaging solution underscores its versatility and value in the food industry.

## 8 Innovations in Cactus‐Based Packaging

### 8.1 Recent Advancements in Biodegradable Film Technologies

Early research focused on leveraging the film‐forming properties of cactus mucilage, a heteropolysaccharide, to develop biodegradable packaging. Initial studies demonstrated that blending mucilage with biopolymers like gelatin and agar improved thermal stability and UV‐light protection, broadening its applicability in food packaging (Gheribi and Khwaldia 2019). Subsequent innovations introduced sodium alginate films enriched with cactus pear peel extract (CPPE), which enhanced tensile strength (1.98 to 3.12 MPa) and reduced water vapor permeability (0.72 to 1.68 × 10^−5^ g h^−1^ m^−1^ Pa^−1^), making them viable for commercial use (Asiri et al. 2024). Further advancements incorporated crosslinking agents such as citric acid to optimize mechanical and barrier properties. Films containing 0.5% citric acid exhibited superior crystallinity and tensile strength, highlighting their potential for food preservation (Kumar et al. 2024). These developments underscore the transition from lab‐scale experiments to scalable, high‐performance materials.

### 8.2 Nanotechnology Applications in Cactus‐Based Films

Nanotechnology has played a crucial role in enhancing the functionality of cactus‐based packaging materials. The incorporation of nanomaterials, such as nanocellulose and halloysite nanotubes, into cactus mucilage films has significantly improved mechanical strength, barrier properties, and bioactive release kinetics (Table 5) (De Medeiros et al. 2024; Gheribi and Khwaldia 2019). Recent studies have shown that nanocellulose‐reinforced films exhibit a dense network structure that reduces gas permeability and enhances tensile strength, making them suitable for active packaging applications (Gheribi and Khwaldia 2019). Additionally, halloysite nanotubes have been incorporated into cactus‐based films to improve their controlled release of antimicrobial agents, further extending the shelf life of packaged food products (De Medeiros et al. 2024). These findings suggest that nanotechnology can significantly enhance the performance of cactus‐based packaging materials, making them viable candidates for commercial applications.

**TABLE 5:** Overview of innovations, challenges, and future directions in cactus‐based packaging.

Characterstics	Key insights	References
Biodegradable film technologies	Cactus mucilage blended with biopolymers improves thermal stability and UV protection. The addition of citric acid enhances tensile strength	Gheribi and Khwaldia (2019), Kumar et al. (2024)
Nanotechnology applications	Nanocellulose and halloysite nanotubes improve mechanical properties, barrier function, and bioactive release	De Medeiros et al. (2024), Gheribi and Khwaldia (2019)
Smart and intelligent packaging	Sensitive films using natural colorants indicate food freshness	De Medeiros et al. (2024)
Health benefits and environmental impact	Biodegradability and antimicrobial activity extend the shelf life of food. Aligns with global sustainability goals	Asiri et al. (2024), Teshager et al. (2024), Van Rooyen et al. (2024)
Scalability and production challenges	Labor‐intensive extraction limits large‐scale adoption. Standardization of extraction techniques is needed	Van Rooyen et al. (2022), De Medeiros et al. (2024)
Market feasibility and cost	High production costs reduce competitiveness against PLA and PHA‐based materials	Olagunju and Kiambi (2024)
Regulatory and consumer adoption	Lack of standardized safety and performance criteria slows market acceptance. Consumer awareness remains low	Tomić et al. (2023), Chavez et al. (2022)
Future directions	Species diversification, improved extraction methods, and enhanced functional properties can drive commercial viability	De Medeiros et al. (2024), Oulabbas et al. (2022)

### 8.3 Smart and Intelligent Packaging Applications

The latest innovations in cactus‐based packaging focus on smart systems that monitor food quality in real time. Building on earlier biocompatibility research, recent studies developed pH‐sensitive films using encapsulated beetroot extract (Table 5). These films shift color from red to yellow in response to pH changes, providing visual indicators of food freshness (De Medeiros et al. 2024). Such intelligent systems not only extend shelf life but also enhance consumer trust by offering real‐time quality assurance.

## 9 Health Benefits and Environmental Sustainability of Cactus‐Based Films

Cactus‐derived films offer significant health benefits in food preservation and packaging, primarily through their biodegradable and antimicrobial properties. These films, made from cactus mucilage and extracts, not only extend the shelf life of fruits and vegetables but also maintain their nutritional and sensory qualities. The incorporation of bioactive compounds further enhances their functional properties, making them a sustainable alternative to conventional packaging materials. Cactus mucilage films help retain vitamins and antioxidants in fruits and vegetables, reducing nutrient loss during storage (De Medeiros et al. 2024). Additionally, films enriched with cactus pear extract exhibit antibacterial and antifungal activities, effectively inhibiting microbial growth (Asiri et al. 2024). Their biodegradability, being composed of natural materials, makes them an eco‐friendly alternative to petroleum‐based plastics (Teshager et al. 2024).

The increasing shift toward cactus‐derived films aligns with global sustainability goals, promoting environmentally friendly packaging solutions (Van Rooyen et al. 2024). With the rising consumer demand for natural and organic products, the market potential for cactus‐based packaging in the food sector is substantial (Mannai et al. 2023). However, challenges such as the standardization of extraction methods and variability in mucilage properties may hinder widespread adoption in the food industry. Addressing these issues is crucial for maximizing their commercial viability.

Cactus‐derived biopolymers offer a promising alternative to petroleum‐based plastics, aligning with global sustainability goals. The integration of antimicrobial agents derived from plants, fungi, and microorganisms can enhance the shelf life and safety of packaged food products (Moeini et al. 2022; Punia Bangar et al. 2021). Additionally, biodegradable and compostable packaging materials derived from renewable sources contribute to environmental sustainability by reducing plastic pollution (Prakoso et al. 2023; Puscaselu et al. 2019).

### 9.1 Safe Dosage Limits for Cactus Mucilage to Mitigate Human Health Risks

Defining universal dosage limits for cactus mucilage remains difficult, as safety depends on factors such as consumption form, concentration, and individual health status. Nonetheless, evidence from both traditional dietary practices and modern clinical research provides useful insights for determining safe intake thresholds. From a dietary perspective, the consumption of fresh or cooked nopal pads ( *Opuntia ficus‐indica* ) is considered safe within normal eating patterns (Griffith 2004). Safety concerns are more relevant when mucilage is concentrated in powders or supplements. Clinical trials have shown that dehydrated nopal powder, at dosages ranging from 1.5 to 5 g/day, effectively regulates blood glucose and lipid levels without adverse events (López‐Romero et al. 2014). For example, a daily intake of 5 g of dehydrated nopal flakes significantly improved glycemic control and cholesterol levels, while maintaining good tolerability (Sánchez‐Uribe et al. 2021).

Preclinical evidence also supports a wide safety margin. Acute toxicity studies in mice reported an LD_50_ exceeding 5 g/kg body weight, equivalent to about 300 g for an adult human with no observable behavioral or lethal outcomes (Arias Gorman et al. 2025; Gebresamuel and Gebre‐Mariam 2012). Human trials further reinforce its safety: daily consumption of 5 g of dehydrated cladode for 6 months showed no negative effects on bone mineral density or body mass index, while long‐term intake of 15 g/day over 2 years was associated with improved bone density (Aguilera‐Barreiro et al. 2013).

Reviews of *Opuntia* spp. highlight that experimental doses tested in animals range from 50 mg/kg to 7 g/kg, with no evidence of genotoxicity or cytotoxicity even at higher levels (Madrigal‐Santillán et al. 2022). However, mild gastrointestinal effects such as diarrhea, nausea, headaches, or increased stool frequency have occasionally been reported with higher intake levels (Madrigal‐Santillán et al. 2022). Traditional dietary use also provides context: consuming 10–17 g/day of prickly pear fruit or 100–500 g/day of roasted cladode material has been associated with chronic metabolic benefits without serious side effects.

## 10 Challenges of Cactus‐Derived Biopolymers

Challenges in the commercialization of cactus mucilage‐based films arise from several factors. One major barrier is the variability in mucilage composition, which is influenced by species differences, extraction methods, and environmental conditions. These inconsistencies hinder the ability to achieve standardized performance, making it difficult to develop uniform products for industrial applications (Oulabbas et al. 2022; Özkır 2023; Velumani et al. 2023). Additionally, scalability and production challenges pose significant obstacles, as labor‐intensive extraction processes and fluctuations in mucilage yield limit large‐scale manufacturing. While techniques such as microwave‐assisted extraction offer potential solutions, they lack standardization, further complicating industrial‐scale implementation (Van Rooyen et al. 2022; De Medeiros et al. 2024). The cost‐effectiveness of cactus‐based films also presents a challenge, as high production expenses, particularly due to the need for additives such as plasticizers and cross‐linkers, make these films less competitive compared to conventional biodegradable polymers like polylactic acid (PLA) or polyhydroxyalkanoates (PHA) (Table 5) (Chavez et al. 2022; Olagunju and Kiambi 2024). Beyond commercialization barriers, functional limitations affect the viability of these films. Their mechanical and barrier properties often fall short of industry requirements, as they may lack the necessary strength, flexibility, or moisture resistance for high‐performance applications (De Medeiros et al. 2024). Another constraint is the dependence on a single species, *Opuntia ficus‐indica* , which restricts global adaptability and sustainability. Diversifying the species used in mucilage extraction could improve the resilience and applicability of these films in various environmental conditions (De Medeiros et al. 2024).

Regulatory and market‐related hurdles further complicate the adoption of cactus mucilage‐based films. The absence of standardized safety and performance criteria makes compliance with food industry regulations challenging, slowing their approval for commercial use (Khezerlou et al. 2021; Tomić et al. 2023). Additionally, consumer acceptance remains a key issue, as limited awareness and skepticism about biodegradable alternatives hinder market adoption. Educating consumers and demonstrating the benefits of these materials could help accelerate their integration into sustainable packaging solutions (Chavez et al. 2022).

## 11 Conclusions and Future Directions

Cactus‐derived biopolymers, particularly mucilage, offer a sustainable alternative to plastic, addressing the growing concern over plastic pollution. With excellent film‐forming, antioxidant, antimicrobial, and UV‐blocking properties, cactus mucilage has potential applications in food packaging, construction, and environmental remediation. Its availability in arid regions and minimal water requirements enhance its sustainability and economic viability. The chemical composition of cactus mucilage consists of polysaccharides with strong water retention, gelling, and mechanical properties. Various extraction methods, such as microwave‐assisted and aqueous extraction, affect yield and functionality, with cactus‐derived films providing superior UV protection and antioxidant activity.

Beyond packaging, cactus mucilage can be used in bio‐admixtures for construction, corrosion inhibition, and water purification. In regions like Ethiopia, cactus‐based biopolymers can reduce plastic reliance, support local industries, and create jobs. On a global scale, they contribute to sustainability by reducing plastic waste, lowering carbon emissions, and promoting a circular economy, aligning with the United Nations Sustainable Development Goals (SDGs), particularly responsible consumption (SDG 12), climate action (SDG 13), and life on land (SDG 15). However, challenges remain, including the need to standardize extraction methods, improve scalability, and reduce costs. Future research should focus on optimizing extraction techniques, enhancing the mechanical and barrier properties of cactus mucilage by blending with other biopolymers and nanomaterials, and developing cost‐effective production methods. Establishing regulatory standards and raising consumer awareness will be essential for market acceptance. New applications in pharmaceuticals, biomedical fields, and smart packaging, such as pH‐sensitive or IoT‐enabled components for real‐time food quality monitoring, should also be explored.

Collaboration among researchers, industries, and policymakers is crucial to overcoming these barriers, ensuring regulatory compliance, and promoting large‐scale adoption. Conducting lifecycle assessments will further validate the environmental and economic feasibility of cactus‐based biopolymers. With continued innovation and investment, cactus‐derived biopolymers can transform the packaging industry, support sustainable development, and contribute to global environmental goals.

## Author Contributions

**Desye Alemu Teferi:** conceptualization (equal), formal analysis (equal), resources (equal), writing – original draft (equal). **Messenbet Geremew Kassa:** supervision (equal), validation (equal), visualization (equal), writing – review and editing (equal).

## Funding

The authors have nothing to report.

## Conflicts of Interest

The authors declare no conflicts of interest.

## Data Availability

This review paper synthesizes information from previously published studies and publicly available data sources. All data and materials referenced in the manuscript are cited appropriately and can be accessed through the original publications or repositories. As this is a review, no new primary data were generated or collected. Consequently, there are no data sets directly associated with this manuscript that require sharing. If specific data sources are not readily accessible, further information can be provided upon request. There are no ethical, privacy, or security concerns associated with the data included in this review.
